# Acute restraint stress rapidly impacts reproductive neuroendocrinology and downstream gonad function in big brown bats (*Eptesicus fuscus*)

**DOI:** 10.1242/jeb.245592

**Published:** 2023-10-12

**Authors:** Mattina M. Alonge, Lucas J. S. Greville, Xuehao Ma, Paul A. Faure, George E. Bentley

**Affiliations:** ^1^ University of California, Berkeley, Department of Integrative Biology, Berkeley, CA 94720-3200, USA; ^2^ Helen Wills Neuroscience Institute, Berkeley, CA 94720, USA; ^3^ McMaster University, Department of Psychology, Neuroscience & Behaviour, Hamilton, ON, Canada, L8S 4L8; ^4^ University of Waterloo, Department of Biology, Waterloo, ON, Canada, N2L 3G1

**Keywords:** Stress, Apoptosis, Hypothalamic–pituitary–gonadal (HPG) axis, Bats, RFamide-related peptide, RFRP, Reproduction

## Abstract

Animals face unpredictable challenges that require rapid, facultative physiological reactions to support survival but may compromise reproduction. Bats have a long-standing reputation for being highly sensitive to stressors, with sensitivity and resilience varying both within and among species, yet little is known about how stress affects the signaling that regulates reproductive physiology. Here, we provide the first description of the molecular response of the hypothalamic–pituitary–gonadal (HPG) axis of male big brown bats (*Eptesicus fuscus*) in response to short-term stress using a standardized restraint manipulation. This acute stressor was sufficient to upregulate plasma corticosterone and resulted in a rapid decrease in circulating testosterone. While we did not find differences in the mRNA expression of key steroidogenic enzymes (StAR, aromatase, 5-alpha reductase), seminiferous tubule diameter was reduced in stressed bats coupled with a 5-fold increase in glucocorticoid receptor (GR) mRNA expression in the testes. These changes, in part, may be mediated by RFamide-related peptide (RFRP) because fewer immunoreactive cell bodies were detected in the brains of stressed bats compared with controls – suggesting a possible increase in secretion – and increased RFRP expression locally in the gonads. The rapid sensitivity of the bat testes to stress may be connected to deleterious impacts on tissue health and function as supported by significant transcriptional upregulation of key pro-apoptotic signaling molecules (Bax, cytochrome *c*). Experiments like this broadly contribute to our understanding of the stronger ecological predictions regarding physiological responses of bats within the context of stress, which may impact decisions surrounding animal handling and conservation approaches.

## Teaser

**Highlighted Article:** The brains of male bats rapidly alter reproductive neuroendocrinology in response to acute stress, with potential negative effects on fertility associated with significant changes in seminiferous tubule morphology and transcriptional upregulation of pro-apoptotic signaling molecules.

## INTRODUCTION

Bats are crucial for the maintenance and stability of many terrestrial ecosystems. Acting as pollinators, seed dispersers, voracious insectivores, and prey for a variety of birds and mammals, the maintenance of bat populations is critical to the success of plants and animals globally. Of the approximately 6500 mammalian species currently recognized, roughly 1400 (21%) are bats. Despite their importance to ecosystem health, relatively little is known about the endocrine and molecular mechanisms regulating the reproductive physiology of bats, including how the reproductive system responds to stress. As anthropogenic change continues and urban sprawl expands, it is important to understand how various acute and chronic stressors impact reproductive signaling and physiology in bats.

Reproduction is an energetically costly process. In nature, animals are often faced with unpredictable challenges that require rapid, facultative physiological responses to support survival (Wingfield, 2015) and that often come at the expense of reproduction (Wingfield and Sapolsky, 2003). Environmental and social stressors can have a negative effect on fertility and/or reproductive outcomes across a variety of vertebrate taxa, including humans (Moberg, 1985; Sapolsky, 1987; Phillips et al., 1989; Knol, 1991; Fenster et al., 1997; Sheiner et al., 2002; Wingfield and Sapolsky, 2003). While the negative relationship between stress and reproduction has been recognized since the earliest studies of stress physiology in a wide variety of classic model species (e.g. Selye, 1939), very little is known about how stress influences the hypothalamic–pituitary–gonadal (HPG) reproductive axis in bats.

Sensitivity and resilience to stress appear to vary both within and among species, even though the basic molecular components of the hypothalamic–pituitary–adrenal (HPA) axis are highly conserved. The endocrine system is essential for regulating circadian rhythms and seasonal energy balance, and it impacts metabolism, cognition, cardiovascular function, reproduction and general behavior collectively as part of preserving the internal milieu (i.e. homeostasis) of the organism (Selye, 1950; reviewed in Sapolsky et al., 2000). The HPA axis is also critical for proper physiological responses following physical or psychological stress. A variety of stressors result in the synthesis and secretion of corticotropin-releasing hormone (CRH; Vale et al., 1981) as well as arginine vasopressin from the paraventricular nucleus and supraoptic nucleus of the hypothalamus, which further stimulates the production and release of adrenocorticotropic hormone (ACTH) from the anterior pituitary gland. Subsequently, ACTH initiates the release of glucocorticoids (GCs; corticosterone and/or cortisol, depending on the species) from the adrenal cortex (Whitnall, 1993; Smith and Vale, 2006; Watts, 2007). These GCs impact cellular activity in a variety of tissues and are subsequently regulated via negative feedback through binding with glucocorticoid receptors (GR) and mineralocorticoid receptors (MR) at the level of the brain and anterior pituitary gland (Reul and DeKloet, 1985; Dallman, 2010; Herman, 2010).

In many species, CRH and adrenal GCs are known to interact directly with the HPG axis. For example, stress-induced increases in CRH and GCs can suppress secretion of luteinizing hormone (LH) and follicle-stimulating hormone (FSH) through actions on gonadotropin releasing hormone (GnRH)-producing neurons and the anterior pituitary gland in rats (Rivier et al., 1986; MacClusky et al., 1988; Petraglia et al., 1987), mice (Jasoni et al., 2005; Phumsatitpong and Moenter, 2018), primates (Dubey and Plant, 1985) and ewes (Ciechanowska et al., 2011), among others (reviewed in Acevedo-Rodriguez et al., 2018; Rivier and Rivest, 1991). Changes within the HPG axis are also associated with alterations in sexual behavior (Retana-Márquez et al., 2003; Bentley et al., 2006) and fertility (Berga and Loucks, 2005; Joseph and Whirledge, 2017). In mammals, peripheral GC injection inhibits the secretion of gonadotropins (LH and FSH) (Dubey and Plant, 1985; Petraglia et al., 1987) and, in males, results in the regression of accessory sex organs, associated with decreased reproductive success with female mates (Lerman et al., 1997). GCs may also exert direct effects on the gonads. In the testes, Sertoli (Hazra et al., 2014), Leydig (Hu et al., 2008) and peritubular cells (Nordkap et al., 2017) are known to express GR. In rats, this receptor-mediated signaling provides a mechanism that is known to impact testes sensitivity to gonadotropins as well as testosterone synthesis (Orr and Mann, 1992). Similarly, female ovaries also express GR with effects on follicle development, sex steroidogenesis and apoptosis (Michael et al., 1993; Yuan et al., 2016; Sasson and Amsterdam, 2003).

The interaction of GCs with the neuropeptide gonadotropin-inhibitory hormone (GnIH) (Kirby et al., 2009) and its mammalian ortholog RFamide-related peptide (RFRP) (Kriegsfeld et al., 2006; Johnson et al., 2007; Murakami et al., 2008) raises questions regarding whether RFRP exerts actions that downregulate the reproductive axis in response to stress, particularly given that GnRH neurons in mammals and birds contact and express receptors for GnIH/RFRP (Bentley et al., 2003; Ubuka et al., 2008; Kirby et al., 2009; reviewed in Son et al., 2022). Acute and chronic stress in male rats causes increased RFRP transcription in the dorsomedial hypothalamus and adrenalectomy prevents this upregulation (Kirby et al., 2009), further supporting the hypothesis that GC signaling is part of the neural RFRP response to stressors. Furthermore, chronic stress in female rats reduces reproductive success, although this can be rescued by knockdown of RFRP (Geraghty et al., 2015). GnIH/RFRP and its receptors are expressed in the steroidogenic cells of the testes and ovaries in birds and mammals, suggesting a role in local regulation of fertility (Bentley et al., 2008; McGuire and Bentley, 2010; Zhao et al., 2010; Squicciarini et al., 2018).

While prior work suggests that acute stress reliably leads to declines in plasma testosterone in some bat species (e.g. Reeder et al., 2004a,b), the mechanisms underlying this effect have not been explored. Furthermore, while there is great interest in understanding the impacts of stressors on the overall activity, life history and population management of bats, little is known about the molecular biology regulating the interactions between the HPA and HPG axes of bat species. Reproduction is a key component supporting individual fitness and, more broadly, the success of wild animal populations, and our study was designed to evaluate the response of the HPG axis to acute restraint stress in male big brown bats (*Eptesicus fuscus*), examining changes in neurobiology, circulating hormone concentrations and downstream gonadal function. We hypothesized that acute stress leads to a decrease in reproductive neuropeptide signaling associated with declines in testosterone production (i.e. fertility). Specifically, we predicted that acute stress will activate the HPA axis, elevate plasma GC concentrations, and decrease plasma testosterone concentrations through changes in steroidogenic enzymes within the gonads. Compared with non-stressed control animals, we predicted stressed bats will exhibit decreased GnRH, increased RFRP immunoreactivity in the brain, and an increased number of RFRP to GnRH neuron contacts, which together would support active inhibition of the HPG axis. The findings from this work provide a necessary foundation for building a more informed framework for ecological predictions regarding how the physiology of bats changes within the context of stressors.

## MATERIALS AND METHODS

### Animals, stress protocol and tissue sampling

Our study used 16 adult male big brown bats, *Eptesicus fuscus* (Beauvois 1796), from a captive, mixed sex, breeding colony housed at McMaster University. Individuals either originated from wild colonies in southern Ontario or were the direct descendants of wild-sourced bats. Animals in the study had a minimum age of 2–5 years. Animal housing included free-flight indoor and outdoor areas where bats experienced natural, seasonal fluctuations in lighting and temperature, and had *ad libitum* access to food (yellow mealworms, *Tenebrio molitor*) and water (as per Skrinyer et al., 2017). Bats selected for the experiment were housed indoors in groups of four in stainless steel wire mesh holding cages [28×22×18 cm l×w×h; ¼-inch (6.35 mm) mesh] for at least 72 h to acclimate to the holding room. Experimental and control manipulations with live animals occurred in June when testosterone and testes size in temperate hibernating bats begin to rise in preparation for spermatogenesis onset (Christian, 1956; Gustafson and Shemesh, 1976; Gustafson and Damassa, 1985). On days of tissue collection, a male bat was removed from each cage and randomly assigned to either the stress or control condition. Bats in the stress condition (*n*=8) were secured ventrum-up for 1 h in a custom restrainer (Ceballos-Vasquez et al., 2014) with their wings outstretched and held in place with Velcro^®^ straps. Body restraint with outspread wings is known to be highly effective in activating a physiological stress response in another small insectivorous bat from North America (Reeder et al., 2004b). Following restraint, bats were deeply anesthetized via isoflurane inhalation and euthanized by decapitation, after which trunk blood was immediately collected. Blood samples were temporarily stored on ice until they were centrifuged (2000 ***g*** for 10 min), after which isolated blood plasma was stored at −80°C for later hormone analysis. Whole brains were extracted and immediately placed in 4% paraformaldehyde for fixation (48–72 h), after which they were cryoprotected (30% sucrose/PBS) before freezing at −80°C. Testes were also removed immediately following euthanasia and were flash frozen on dry ice prior to −80°C storage. Control bats (*n*=8) were anesthetized via isoflurane inhalation and decapitated immediately after removal from the home cage, and tissue collection followed identical protocols to the stress condition, with blood collection occurring within 3 min of removal from their home cage (mean: 82 s, range: 71–93 s). All procedures were approved by the Animal Research Ethics Board of McMaster University and conformed to the Guide to the Care and Use of Experimental Animals published the Canadian Council on Animal Care.

### Hormone analysis

Circulating plasma corticosterone concentrations were quantified using a commercially available enzyme-linked immunosorbent assay (ELISA) kit (501320, Cayman Chemical, Ann Arbor, MI, USA). Control plasma was diluted 1:160 (sample:kit buffer) and stress plasma was diluted 1:600 prior to plating. Percentage binding for all samples was between 32% and 53% and fell within the standard curve range for this kit (8.2–5000 pg ml^−1^, fit=98%) with coefficient of variation (CV) for all samples assayed ranging between 0.99% and 3.68%. Reported kit cross-reactivity with cortisol provided by the manufacturer is 2.5% (501320, Cayman Chemical). Circulating plasma testosterone concentrations were also determined with a commercially available ELISA kit (ADI-901-065, Enzo Life Sciences, Farmingdale, NY, USA), with control plasma diluted 1:640 and stress plasma diluted 1:100. Percentage binding for individual testosterone samples ranged from 32% to 86% and fell within the standard curve range for this kit (7.8–2000 pg ml^−1^, fit=98.6%) with CV for all samples ranging between 0.66% and 5.88%. Prior to sample processing, both kits were validated for use in *E. fuscus* by testing for parallelism of serially diluted plasma pools for bats from stress and control treatments (*n*=8 control samples, *n*=8 stress samples) alongside the kit standard and recommended standard curve dilution series. For the corticosterone kit, pooled plasma was diluted 1:137–1:880 for stress samples and 1:20–1:160 for control samples. For the testosterone kit, pooled plasma was diluted 1:20–1:640 for both control and stress samples. For both hormones, results of the parallelism test confirmed that the target analyte was recognized by kit reagents in a predictable, dose-dependent manner. It was possible for all samples (total *n*=16 animals) to be assayed on one plate per hormone and thus no inter-assay variation was required to be calculated.

### Double-label immunohistochemistry – RFRP and GnRH immunoreactivity

Brains were sectioned (40 µm) with a cryostat and tissue slices were organized into three parallel series. Sections were immediately transferred to antifreeze solution (5.47 g sodium phosphate dibasic, 1.59 g sodium phosphate monobasic, 9 g NaCl, 10 g PVP-40, 300 g sucrose, 300 ml ethylene glycol, 10 ml 0.1 mol l^−1^ sodium azide, in 1 l dH_2_O) for storage at −20°C prior to immunohistochemistry. Six brains from each treatment group were sectioned coronally, while sagittal sections were collected from the remaining two brains per treatment group in order to maximize visibility of target neuropeptide fiber reactivity and connectivity across brain regions. Sections were washed 5 times with 1× PBS (pH 7.4) prior to incubating in 0.5% hydrogen peroxide in PBS for 15 min at room temperature. After washing again in PBS, a blocking solution of 2% normal goat serum (NGS) in PBS/0.2% Triton X-100 (PBST) was added, and sections were gently agitated for 2 h at room temperature. To quantify the number of cells containing GnRH, tissue sections were incubated in a solution containing anti-GnRH primary antibody (HU60, gift from H. Urbanski, Oregon Health and Science University, 1:10,000 in 0.2% PBST/1% NGS) for 48 h at 4°C. This was followed by incubation in goat anti-rabbit biotinylated secondary antibody (1:200 in PBST) before Vectastain Elite ABC (PK-6100, Vector Laboratories Inc., Newark, CA, USA), and color development of GnRH immunoreactive (GnRH-ir) material was performed using Vector DAB substrate (SK-4100, Vector Laboratories Inc.) as per the manufacturer's instructions. Sections were washed 3 times in 0.2% PBST, then incubated in a solution of anti-GnIH primary antibody (PAC123,124 1:5000 in 0.2% PBST/1% NGS) for 48 h at 4°C to simultaneously label cells containing mature RFRP peptide. Following primary antibody incubation, sections were transferred to goat anti-rabbit biotinylated secondary antibody solution (1:200 in PBST) before Vectastain Elite ABC, and color development of RFRP immunoreactive material was performed using Vector VIP substrate (SK-4600, Vector Laboratories Inc.). The specificity of the anti-GnIH (white-crowned sparrow) antibody in the big brown bat was previously validated in our lab using a standard pre-adsorption method with GnIH peptide, which resulted in a complete loss of immunoreactivity, as expected (see Alonge et al., 2022). The anti-GnRH antibody used in our study (HU60) has been used extensively in other studies within our group and beyond, with successful and accurate detection across a wide range of ‘traditional’ and less classic model species (Ferris et al. 2015; McGuire et al., 2013; Dixit et al., 2022), and for our study we compared our anti-GnRH labeling with that of a no-HU60 antibody control to validate technical steps of our immunohistochemistry protocol. The total number of GnRH- and RFRP-immunoreactive cell bodies was counted separately across one entire series of sections for each bat brain, including both coronal and sagittal brain sections, by individuals blind to the experimental treatment. One bat from the stress group was excluded from analysis of the RFRP cell number data because of a problem during the immunolabeling protocol which meant we were unable to process the tissue.

### RNA isolation and quantitative PCR

One half of one testis from each male bat was used for RNA isolation, conducted using 500 µl PureZOL (7326890, Bio-Rad, Hercules, CA, USA) in an RNase-free snap-cap tube. Tissue was homogenized over ice before adding PureZOL to 1.0 ml total volume for RNA recovery, following the manufacturer's instructions. Samples were eluted in 30 µl nuclease-free water. RNA integrity was determined for all samples (Agilent Bioanalyzer, Functional Genomics Laboratory, UC Berkeley, Berkeley, CA, USA) with resulting RIN >7. For each sample, 1 µg RNA was converted to cDNA (iScript cDNA synthesis kit 1708890, Bio-Rad) which was then diluted 1:5 with nuclease-free water as a working stock solution that was stored at −20°C until use. All primers for reference genes and genes of interest (GOI) (Table 1) were validated for amplification of a single product in big brown bat testes using endpoint PCR (Platinum Taq High Fidelity 11304-011, Invitrogen, Waltham, MA, USA) and visualized on an agarose gel. Primer efficiencies were determined using a 4-fold serially diluted five-point standard curve for all gene targets made from pooled cDNA from bat testes; calculated efficiencies fell within a range 90–110% for all primer pairs used. The cDNA samples from each individual bat testis were used at 1:20 dilution for quantitative PCR (qPCR) for all genes except *β-actin*, which was used at 1:80 dilution. Reactions were prepared following the protocols provided by the kit (SsoAdvanced SYBR Green Supermix 1725271, Bio-Rad) using primers at a final concentration of 0.5 µmol l^−1^ per reaction. Absence of contamination was confirmed by lack of amplification of no-template (no cDNA) and no-reverse transcriptase controls during qPCR. Amplification of a single product was verified by the final dissociation/melting curve. Relative mRNA expression was calculated using the ΔΔCt (Livak) method (Livak and Schmittgen, 2001) using two reference genes (*GAPDH* and *β-actin*) confirmed to be unaffected by stress treatment. GOI were selected based on critical roles in GC sensitivity (e.g. GR, MR), a broad inhibitory role within the HPG axis (e.g. RFRP) and pro-apoptotic signaling (e.g. Bax, Apaf-1, cytochrome *c*; Fig. 6D). Because the pathway associated with sex steroid synthesis is layered and complex, we opted to begin by selecting gene targets that directly influence testosterone production or conversion (e.g. aromatase, 5-alpha reductase) as well as a critical rate-limiting step (steroidogenic acute regulatory protein, StAR). These data reflect potentially rapid transcriptional changes within the gonads in response to our stress treatment.

**Table 1.**
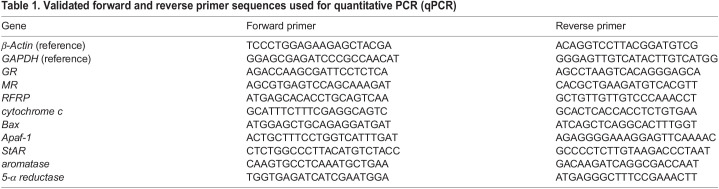
Validated forward and reverse primer sequences used for quantitative PCR (qPCR)

### Testes histology, morphology and TUNEL labeling

To accompany transcriptional analysis of pro-apoptotic genes, we sought to further explore potential damaging effects on the gonads using terminal deoxynucleotidyl transferase dUTP nick end labeling (TUNEL), a histological technique that detects late-stage apoptotic DNA damage. A subset of control (*n*=5) and stress treatment (*n*=4) bats provided sufficient testis tissue to use for cryosectioning and hematoxylin and eosin Y (H&E) staining. Slides were prepared with a series of three 25 µm thaw-mounted testis sections from individual bats and fixed for 10 min in 4% paraformaldehyde before proceeding to the standard protocol for H&E staining. Images were captured at ×200 total magnification and imported into ImageJ where the diameter of each circular seminiferous tubule in cross-section was measured by drawing a straight line from basement membrane to basement membrane across the center of the tubule; all circular tubules were measured within each image. Mean seminiferous tubule diameter (µm) for each individual bat was determined by converting the unitless ImageJ output measures into µm using a calibrated scale bar (1.32 raw ImageJ units=200 µm) and calculating the average for 16 total tubules per individual. Seminiferous tubular diameters of bats from the stress and control groups were compared with Welch's *t*-test.

A series of sections (16 µm) was collected from one testis of each bat and thaw-mounted directly on to slides for quantifying the amount and localization of TUNEL-positive cells. Because of limited tissue samples remaining from the animals in the study, a subset of control (*n*=6) and stress (*n*=5) bats were processed for TUNEL analysis. Slides were stored at −20°C until processing. Following the protocol supplied with a commercially available kit (TACS 2Tdt-DAB In Situ Apoptosis Detection Kit 4810-30-K, R&D Systems Inc., Minneapolis, MN, USA), sections were removed from −20°C and allowed to dry completely overnight and equilibrate to room temperature before proceeding to labeling. Sections were then gently rehydrated through a graded series of ethanol incubations (100%, 95%, 70%) and fixed on slides in 4% paraformaldehyde/PBS for 10 min at room temperature. Slides were then washed in 1× PBS for 10 min and the sections were permeabilized in cytonin solution (4876-05-01, R&D Systems Inc.) for 45 min at room temperature inside a humidity box. Tissue was washed in distilled water 2 times (5 min each) before endogenous peroxidase activity was quenched in a 1:10 solution of 30% hydrogen peroxide in methanol for 5 min. A positive control slide was generated by treating tissue with TACS nuclease for 10 min at room temperature and then processed alongside the experimental slides after this point. All slides were washed in 1× PBS and incubated in 1× TdT labeling buffer (provided by the kit) for 5 min before adding TdT enzyme reaction solution. Tissue was incubated with this reaction mix for 1 h in a humidity box at 37°C. After incubation with enzyme, tissue was incubated in the provided Stop Solution for 5 min and subsequently washed in 1× PBS 2 times (5 min each). Following the kit instructions, a strep-horseradish peroxidase (HRP) solution was prepared and added to tissue for 10 min at 37°C, after which slides were thoroughly washed in 1× PBS to remove excess antibody and minimize background labeling. Color development of TUNEL-positive immunoreactive material was performed using Vector DAB substrate (SK-4100, Vector Laboratories Inc.). Tissue was counterstained using 1% Methyl Green and dehydrated in a graded ethanol series (70%, 95%, 100%) before clearing in Histoclear (5 min) and cover slipping using Permount medium (SP15-500, Thermo Fisher, Waltham, MA, USA).

Microscopy images (×400 total magnification, Zeiss Imager A1 and AxioCam MRC5) were collected and used for TUNEL analysis. Eight images were collected per individual bat and the total number of cells (via Methyl Green staining) was quantified using Fiji. Within the software, each 300ppi microscope image was converted to 16-bit grayscale, background subtracted (value=40 standardized across all images), and threshold adjusted to highlight cells. Particle analysis was modified such that only particles greater than 180-pixel units were counted with standardized degree of circularity (0.3–1.0, where 1.0 is a perfect circle), and the total number of particles (i.e. cells) within each image was automatically counted. Following this, the number of TUNEL-positive cells within each image was manually counted by volunteers blind to treatment (ImageJ, multipoint tool) and the percentage of TUNEL-positive cells was calculated per image. From this, a mean percentage of TUNEL-positive cells was calculated for each individual based on the cumulative data across 8 images per bat.

### Statistical analysis

All data are reported as the mean±s.e.m. No individuals were excluded from statistical comparisons of treatment (stress) and control groups unless otherwise noted below. Prior to all analyses of treatment-level differences, a Shapiro–Wilk test was used to evaluate the normality of data for bats in the stress and the control groups. Homogeneity of variances among the groups was evaluated using Fligner–Killeen test, which is appropriate when data are non-parametric. When data were normally distributed and homoscedastic, a two-sample Welch's *t*-test was used to compare the means of treatment and control groups; for each comparison, the test statistic, degrees of freedom and associated *P*-value are reported. In cases where the data were not normally distributed and/or variances were heteroscedastic, a Wilcoxon rank sum test was used to compare the treatment and control groups; for each comparison, the associated *W*- and *Z*-values are reported along with calculated *P*-values. Effect size (*r*) for rank sum tests was also calculated using the *statix* package in R where *r*=*Z*/√*N* (where *N* is the sample size). Statistically significant differences between treatment and control groups (*P*<0.05) are indicated in the figures. We used Pearson's correlation coefficient (*R*) to evaluate the linear relationship between two variables, with the direction/strength, and significance reported through *R-* and *P*-values, respectively. In the case of analyzing the relationship between RFRP and GR expression (see below), one stress-treated individual was excluded from the analysis as an outlier (RFRP mRNA relative expression value=14.41; >1.5× IQR); however, inclusion of this individual does not change the significant relationship detected (*R*=0.6, *P*=0.016). To aid in the interpretation of our correlational data, we conducted power analysis via the *pwr* package in R using sample size (*n*), medium effect size (0.50) and significance value (0.05) as inputs. Based on this analysis, we found the following: in cases where *n*=16 (treatment not included in the model), powe*r*=0.529; in cases where *n*=8 (treatment had a significant effect and was included in the model), powe*r*=0.255.

## RESULTS

### Acute restraint rapidly activates the HPA axis, decreases plasma testosterone and alters GC sensitivity of bat testes

**Fig. 1.**
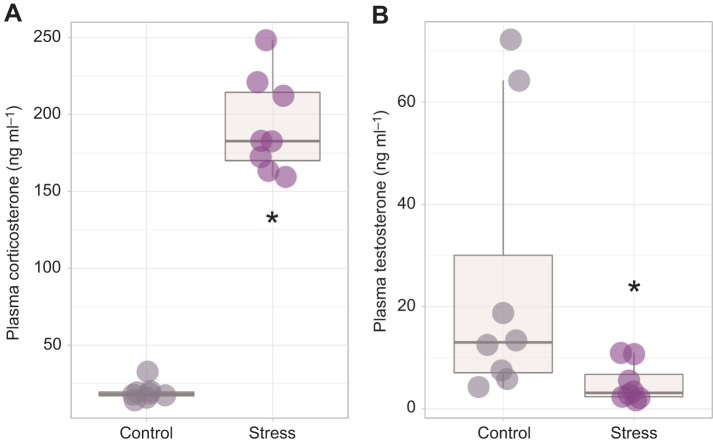
**Impact of 60 min of restraint stress on circulating corticosterone and testosterone concentration in big brown bats.** Stress significantly upregulated plasma corticosterone (A) and resulted in significantly reduced testosterone (B) in male bats (*n*=8) compared with control animals (*n*=8; *P<0.05). Boxplots display the group median (horizontal line), the interquartile range (box limits, 25–75%) and minimum and maximum values (vertical lines); outliers are displayed as stand-alone points beyond the vertical lines.

**Fig. 2.**
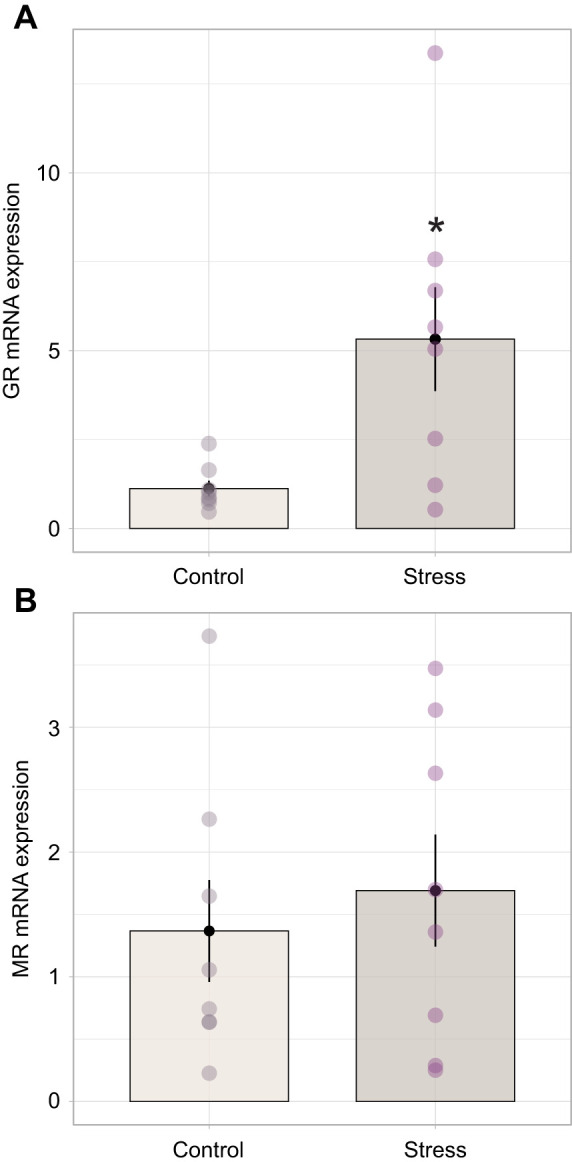
**Impact of 60 min of restraint stress on testes receptor gene expression.** (A) Glucocorticoid receptor (GR) mRNA expression was significantly higher in bats in the stress condition (*n*=8) compared with control animals (*n*=8; *P<0.05). (B) There was no effect on mineralocorticoid receptor (MR) expression. Bar plots represent means±s.e.m., with individual expression depicted within each group by circles to highlight interindividual variation and range.

Continuous restraint for 60 min resulted in a significant increase in circulating plasma corticosterone concentration compared with controls (*W*=0, *Z*=−3.31, *P*=0.00093, *r*=0.84; Fig. 1A), thereby validating the efficacy of the restraint protocol in activating the HPA axis. Bats experiencing acute restraint stress also had significantly reduced plasma testosterone compared with control animals (*W*=57, *Z*=−2.57, *P*=0.01, *r*=0.66; Fig. 1B). The endocrine response to restraint was accompanied by an approximately 5-fold increase in GR mRNA transcription in the bat testes (*W*=9, *Z*=−2.43, *P*=0.014, *r*=0.60; Fig. 2A), although no change in MR expression was observed (*W*=27, *Z*=−0.46, *P*=0.645, *r*=0.13; Fig. 2B).

### Steroidogenic enzyme mRNA expression is unchanged in the bat testes following acute restraint, but stressed bats exhibit decreased seminiferous tubule diameter

**Fig. 3.**
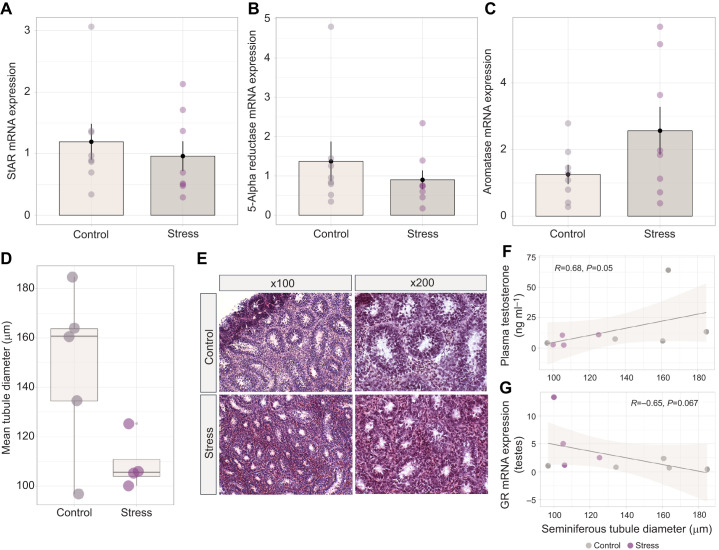
**Expression of key steroidogenic enzymes in the bat testes and impact of stress on seminiferous tubule diameter.** No significant transcriptional effect of acute restraint stress was observed for (A) steroidogenic acute regulatory (StAR) protein, (B) 5-alpha reductase or (C) aromatase. Bar plots represent means±s.e.m., with individual expression depicted within each group by circles to highlight interindividual variation and range. (D) Individual mean seminiferous tubule diameter of control (*n*=5) and stress-treated (*n*=4) groups. Boxplots display the group median (horizontal line), the interquartile range (box limits, 25–75%) and minimum and maximum values (vertical lines); outliers are displayed as stand-alone points beyond these vertical lines. (E) Representative testes microscope images following hematoxylin and eosin staining. (F) A significant positive relationship was detected between individual plasma testosterone concentration and seminiferous tubule diameter using Pearson's correlation, while (G) individuals with greater mRNA expression for gonadal GR tended to have a smaller seminiferous tubule diameter. Shaded region represents 95% confidence interval.

Despite a significant reduction in circulating testosterone with stress, we did not detect transcriptional changes in enzymes associated with testosterone regulation. Specifically, stress did not alter the relative mRNA expression of StAR protein (*W*=40, *Z*=−0.788, *P*=0.430, *r*=0.21; Fig. 3A), 5-alpha reductase (*W*=42, *Z*=−0.977, *P=*328, *r*=0.26; Fig. 3B) or aromatase (d.f.=9.25, *P*=0.122, *r*=0.85; Fig. 3C) in the testes of *E. fuscus*, demonstrating that rapid declines in testosterone were not a result of changes in expression of these steroidogenic enzymes. Seminiferous tubule diameter tended to be smaller in stress-treated versus control bats (group means were found to be 109.14 μm and 148.08 μm, respectively), although the difference was not quite significant (*P*=0.059, d.f.=5.0273, *t*=2.4223, *r*=1.46; Fig. 3D,E). Individuals with higher concentrations of plasma testosterone exhibited larger seminiferous tubule diameters (*R*=0.68, *P*=0.05; Fig. 3F). There was a moderate negative correlation between GR mRNA expression in the testes and seminiferous tubule diameter that was not significant (*R*=−0.65, *P*=0.067; Fig. 3G).

### Acute restraint stress reduces RFRP immunoreactivity in the bat brain and upregulates RFRP expression in the testes

**Fig. 4.**
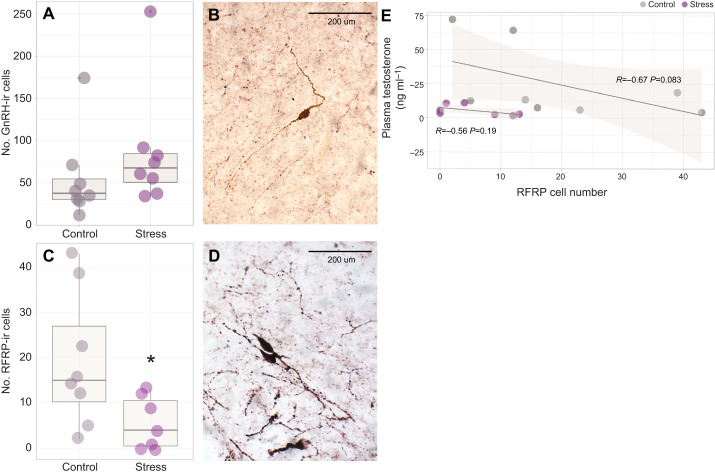
**Impact of 60 min of restraint stress on key stimulatory and inhibitory reproductive neuropeptides.** (A) The total number of immunoreactive (ir) cell bodies labeled for gonadotropin-releasing hormone (GnRH) in the brains of male bats was unchanged by acute restraint (*n*=8 control, *n*=8 stress treated). (B) A representative image of GnRH-positive cells labeled using Vector DAB substrate (viewed at ×400 magnification). (C) However, significantly fewer RFamide-related peptide (RFRP)-immunoreactive cell bodies were observed in stressed animals (*n*=7) compared with controls (*n*=8; *P<0.05). (D) A representative image of RFRP-positive cells labeled using Vector VIP substrate. Boxplots display the group median (horizontal line), the interquartile range (box limits, 25–75%) and minimum and maximum values (vertical lines); outliers are displayed as stand-alone points beyond these vertical lines. (E) We found no significant within-individual relationship between quantified RFRP-ir cell number and the respective concentration of plasma testosterone, although there was a tendency (*P*=0.083) within control animals for those with a greater RFRP cell number to exhibit lower circulating testosterone. Shaded region represents 95% confidence interval.

**Fig. 5.**
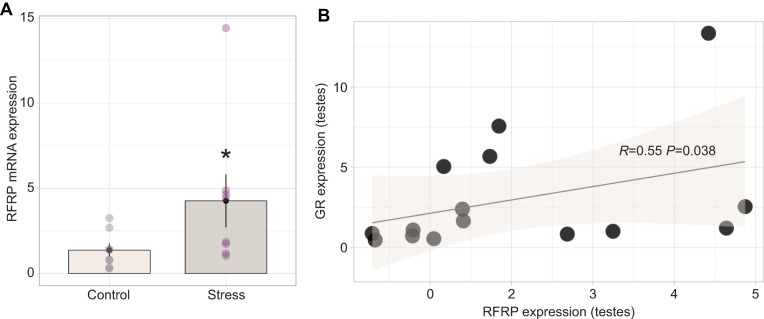
**Impact of 60 min of restraint stress on testes RFRP regulation and relationship with GR expression.** (A) Acute restraint stress resulted in transcriptional upregulation of RFRP in the testes (*n*=8 control, *n*=8 stress treated; *P<0.05). Bar plots represent means±s.e.m., with individual expression depicted within each group by circles to highlight interindividual variation and range. (B) Increase in RFRP mRNA expression was positively correlated with within-individual testes GR expression, with no apparent effect within treatment groups (*P*>0.05). Shaded region represents 95% confidence interval.

Histological localization of hypothalamic cell bodies containing immunoreactive GnRH and RFRP peptides measured with floating immunohistochemistry revealed that the number of GnRH-ir cells did not differ between stress-treated and control bats (*W*=16, *Z*=−1.62, *P*=0.104, *r*=0.42; Fig. 4A). In contrast, there was a significant decrease in the number of RFRP-ir cell bodies in bats subject to restraint stress (d.f.=9.21, *P*=0.039, *r*=1.21; Fig. 4C). Relative RFRP mRNA expression was significantly upregulated within the testes of stress-treated compared with control bats (*W*=12, *Z*=−2.06, *P*=0.037, *r*=0.52; Fig. 5A). Although there was no correlation between within-individual RFRP expression and plasma testosterone concentration in either the stress-treated (*R*=−0.56, *P*=0.19) or control groups (*R*=−0.67, *P*=0.083), within the control group, bats with greater RFRP cell numbers tend to exhibit lower concentrations of plasma testosterone (Fig. 4E). There was a positive correlation between within-individual GR expression and RFRP expression (*R*=0.55, *P*=0.038; Fig. 5B).

### Rapid upregulation of apoptotic gene expression in the gonads but no significant difference in TUNEL-positive cell number

**Fig. 6.**
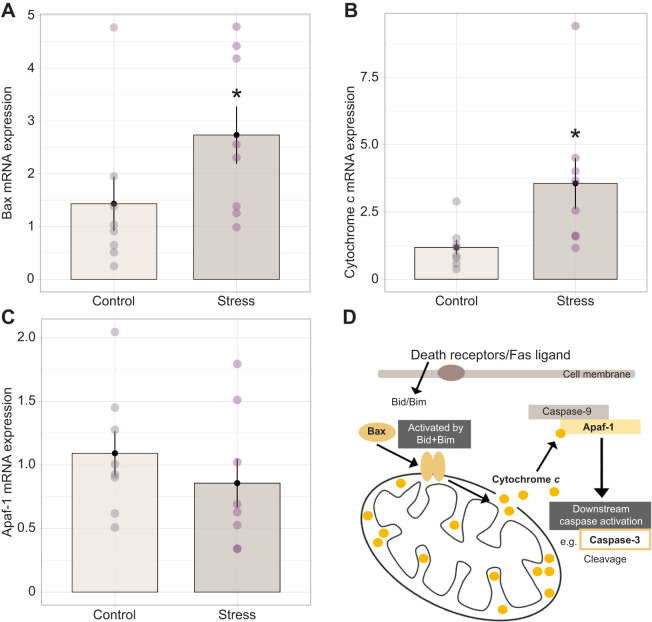
**Effect of 60 min of restraint stress on pro-apoptotic mRNA expression in the testes.** In bats exposed to restraint (*n*=8) there was a significant transcriptional upregulation of (A) Bax and (B) cytochrome *c*, but not (C) Apaf-1 mRNA expression relative to control animals (*n*=8; *P<0.05). Bar plots represent means±s.e.m., with individual expression depicted within each group by circles to highlight interindividual variation and range. (D) These target molecules are involved in a mitochondria-mediated pro-apoptotic pathway that begins with activation of death receptors in the cell membrane. This signal of cellular stress stimulates Bid and Bim to activate Bax, which then dimerizes and induces mitochondrial membrane permeabilization and subsequent release of cytochrome *c* from the outer mitochondrial matrix. Cytochrome *c* binds to Apaf-1, permitting the interaction of Apaf-1 with caspase-9. Activation of caspase-9 is the first in a series of classical downstream caspase activation steps that ultimately leads to cellular apoptosis.

**Fig. 7.**
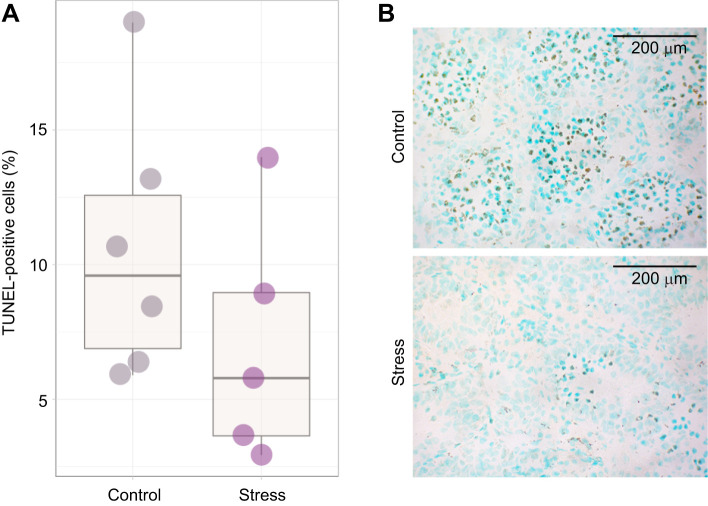
**No effect of stress on late-stage apoptosis in the bat testes.** (A) There was no difference in the mean percentage of total TUNEL-positive cells (indicating apoptosis) in the testes of stressed (*n*=5) bats compared with those in the control (*n*=6) group. Boxplots display the group median (horizontal line), the interquartile range (box limits, 25–75%) and minimum and maximum values (vertical lines); outliers are displayed as stand-alone points beyond these vertical lines. (B) There was a tendency for control bats to exhibit a greater percentage of TUNEL-positive cells. Individual cell nuclei were visualized (at ×400 magnification) by Methyl Green stain and TUNEL-positive cells with Vector DAB substrate (brown).

There was a significant upregulation of *Bax* (*W*=13, *Z*=−1.96, *P*=0.049, *r*=0.50; Fig. 6A) and *cytochrome c* (*P*=0.0069, *W*=7, *Z*=−2.69, *r*=0.66; Fig. 6B) mRNA expression in the testes following 60 min of restraint stress; however, no change in *Apaf-1* expression was observed (*P*=0.441, *W*=40, *Z*=−0.76, *r*=0.21; Fig. 6C). Despite rapid changes in mRNA expression, there was no difference in the number of TUNEL-positive cells between the stress-treated and control groups (*P*=0.2469, *t*=1.2396, d.f.=8.87, *r*=0.74; Fig. 7A), although there was a tendency for control bats to exhibit an overall higher percentage of TUNEL-positive cells in the testes (Fig. 7B). Finally, there was no correlation between within-individual degree of mRNA expression and the number of TUNEL-positive cells for any genes of interest (statistics for each gene not shown).

## DISCUSSION

We used a holistic approach to explore the impacts of acute stress on the reproductive physiology of male bats by examining changes in key reproductive neuropeptides (GnRH and RFRP), circulating plasma hormones (corticosterone and testosterone), and downstream changes in testes gene expression associated with gonad health and fertility. Acute restraint stress resulted in changes in RFRP expression at both the hypothalamic and gonadal levels of the HPG axis. This was associated with a predictable decline in circulating testosterone, but no change in mRNA expression of key steroidogenic enzymes (StAR, aromatase, 5-alpha reductase) in the testes. Morphological differences in the gonads were observed between stress-treated and control animals, with the seminiferous tubule diameters of stressed bats smaller than those of control bats. In contrast, there was no apparent effect of restraint stress on cellular GnRH immunoreactivity in the bat brain. Stress likely increased gonadal sensitivity to GCs through a rapid 5-fold increase in GR expression. Whether mediated by neurobiological signaling or direct GC effects on the gonads, we also found evidence for rapid pro-apoptotic gene expression changes in the testes in response to acute (60 min) restraint stress. Together, our findings suggest that RFRP may be involved in a broad downregulation of the HPG axis in male bats exposed to acute stress and support the possibility that the testes may receive direct endocrine signals that influence fertility at the sex steroid and cellular health levels.

Previous descriptions of GC responses in bats have focused largely on stress associated with handling and manipulating individuals. For example, in captive variable flying foxes (*Pteropus hypomelanus*), restraint during handling resulted in a 4-fold rise in ACTH concentration after 1 h, with cortisol becoming significantly elevated after only 20 min and peaking at levels almost 800% greater than baseline measurements (Widmaier and Kunz, 1993; Widmaier et al., 1994). Similar patterns of GC dynamics have been reported for *Pteropus vampyrus* and *Pteropus pumulis* following up to 1 h of restraint stress (Reeder et al., 2004a, 2006a; Widmaier et al., 1994). Our experiment was designed specifically to examine impacts of restraint stress on the HPG and HPA axes, so we used these previous studies to determine the appropriate time for sampling tissues to quantify physiological changes associated with HPA activation. Our within-species experiment and design produced a clear and reliable time frame for detecting neuroendocrine and gonadal changes in response to a highly standardized acute stressor. While a dramatic increase in plasma corticosterone provided sufficient validation that our manipulation activated the HPA axis, it is important to acknowledge that cortisol may also be an important mediator of metabolic and stress physiology in bats and it is possible that these two GCs may have actions and effects independent of one another which we cannot say from our study alone. Despite cortisol being previously posited as the ‘main’ GC in a selection of bat species examined – with a tendency to be found at higher concentrations – corticosterone does exhibit a pronounced increase in response to restraint stress as seen in our study. We encourage further work to elucidate not only specific signaling pathways that are activated by increases in circulating corticosterone but also whether predicted parallel shifts in cortisol exert differing effects or show a relationship to other facets of reproductive downregulation that we targeted for this experiment which were not significant for corticosterone.

### Acute restraint is sufficient to induce changes in reproductive neuroendocrinology

Stress-induced decreases in androgen concentrations have been observed in other bats (Reeder et al., 2004a, 2006a), although neither the mechanism(s) underlying the declines nor the implications for reproductive success have been examined. Our data indicate that 60 min of restraint stress is sufficient to induce neurobiological as well as gonadal changes within the HPG axis. Although restraint stress did not influence the number of GnRH-ir cells in the brain, there was a decrease in the number of RFRP-ir cells. While this may seem counterintuitive given the inhibitory role of RFRP within the reproductive axis, it may reflect active secretion of this neuropeptide in response to the restraint treatment. This is an interesting contradictory finding as acute immobilization stress increases the number of hypothalamic RFRP-ir cells in rats (Kirby et al., 2009) and the number of GnIH-ir cells in birds (Calisi et al., 2008), with the latter varying by season. In sheep, acute restraint stress did not affect concentrations of RFRP mRNA or mature peptide in the brain (Papargiris et al., 2011), but the same group later found that the number of close contacts (via immunohistochemical methods) between RFRP-ir fibers and GnRH cell bodies increased following acute stress (∼10% of cells in the control group versus ∼40% after stress) and increased the number of RFRP cells co-labeled with Fos (Clarke et al., 2016). Without being able to measure RFRP release into the bat hypothalamo-pituitary portal system, it is difficult to determine the dynamics of RFRP release in response to our manipulation. It is possible that in *E. fuscus*, GnRH may be more important for regulation of seasonal reproduction and less involved than RFRP in facultative responses to an unpredictable, acute stressor.

In our study, GnRH-ir cell bodies were more abundant than RFRP-ir cells, regardless of treatment. In agreement with our data, previous studies in other taxa have revealed a lack of change in GnRH, even in response to a chronic stress paradigm (Du Ruisseau et al., 1979; Kirby et al., 2009). Our results may reflect the inhibitory actions of RFRP secretion on GnRH cells; such effects would prevent subsequent GnRH release without changing GnRH-ir cell number and would ultimately inhibit LH and FSH secretion, thereby reducing testosterone production (as seen in Ducret et al., 2009; Gojska et al., 2014). Repeated cortisol injection (used to mimic chronic stress pharmacologically) increases the number of neuron–fiber contacts between RFRP and GnRH cells in the sheep brain (Clarke et al., 2016), but the functional consequences of this change have yet to be identified. While we did not quantify the number of putative connections between RFRP-ir fibers and GnRH cell bodies in our study design, this would be a feasible next step and successful tracing of RFRP–GnRH contacts would help elucidate the mechanisms regulating potential shifts in downstream HPG axis functions.

In mammals (Kirby et al., 2009; Gojska and Belsham, 2014) and birds (Calisi et al., 2010; Son et al., 2014), RFRP-producing cells are known to express GR and there is putative molecular evidence for transcriptional upregulation of GnIH/RFRP under stress through GC-stimulated recruitment of GR to the GnIH promoter region (Son et al., 2014). As a result, GC signaling to RFRP-producing cells in the hypothalamus may trigger the production and/or secretion of RFRP, leading to subsequent suppression of GnRH release. This may inhibit the synthesis or secretion of gonadotropins into the circulation which could influence the downstream function of Leydig and Sertoli cells of the male gonads, including spermatogenesis and sex steroid production (Shalet, 2009), as reflected in our data. As we continue to broaden the techniques and tools validated for use in bat species, future studies should attempt to examine potential changes to LH and FSH concentrations in response to various stressors to strengthen our foundational understanding of how physiological challenges impact the reproductive endocrinology of bats.

### Glucocorticoid sensitivity increases rapidly at the level of the bat gonad

In the present study, we demonstrate that stress-induced declines in the concentration of circulating testosterone may be linked to negative impacts on male bat fertility at the level of the testes. Our data show that acute stress can impact male gonads directly, resulting in rapidly increased transcription of GR and RFRP. The approximate 5-fold increase in GR expression we observed indicates the onset of a heightened sensitivity to GCs which, depending on the cell type, may impact downstream testosterone secretion. Furthermore, it has been shown in mice that *in vitro* application of RFRP to isolated testes results in the simultaneous reduction in spermatogenesis and germ cell proliferation (Anjum et al., 2014), which suggests that the upregulation of RFRP expression we observed in the bat testes may directly affect male bat fertility in a similar fashion. Furthermore, Orr and Mann (1990) found that the restraint stress-induced decrease in testosterone in rats was LH independent. It is possible that the decrease in plasma testosterone we observed in bats occurs through direct action of GCs on Leydig cells via GR, a mechanism known to suppress testes sensitivity to gonadotropins and dampen testosterone production in rats (Orr and Mann, 1992). The upregulation of GR mRNA expression we observed in the bat gonads provides some support for this, although we cannot say specifically what cell types within the testes may be altering their transcriptional activity.

Seasonal or life history stage-dependent GC responses to stress have been observed in other taxa (McGuire et al., 2013; Vera et al., 2011; Wingfield et al., 1992), including bats (Gustafson and Belt, 1981; Klose et al., 2006; Reeder et al., 2004b). Sex differences in HPA activation have been observed in *P. hypomelanus*; while males displayed no detectable impact of handling on GC levels, reproductive females were characterized by differential responses to stress throughout gestation (Reeder et al., 2004a). Glucocorticoid concentrations in little brown myotis (*Myotis lucifugus*) are generally higher in females, with both sexes showing elevations during autumn swarming (i.e. the primary reproductive season), and reproductive females showing elevated baseline levels during mid-to-late pregnancy in late spring (Reeder et al., 2004b). Although we were unable to characterize the sensitivity of the HPG axis to stress across annual seasons or between sexes in our study, it is possible that highly seasonal species such as *E. fuscus* (that only reproduce once per year) may exhibit different responses to stress during their annual reproductive cycle or even between early versus late periods of reproductive investment (i.e. May to September). Because stress and RFRP have broad links to suppression of sexual behavior (Retana-Márquez et al., 2003; Dubey and Plant, 1985; Sato et al., 1996), it is possible that the stress-induced changes in reproductive neuroendocrinology we observed may lead to further organism-level changes in mating behavior or individual reproductive success, which could have negative fitness impacts.

### Acute restraint significantly impacts testes morphology and upregulates pro-apoptotic gene expression

We found no changes in mRNA expression of steroidogenic enzymes in the testes (StAR, aromatase, 5-alpha reductase); however, there was a decrease in seminiferous tubule diameter in stressed compared with non-stressed bats, which suggests a rapid regressive tissue response. It is possible that other enzymes in the testosterone synthesis pathway (e.g. cytochrome p450) may respond to acute restraint in bats, or that functional enzyme activity may be altered, thus providing a causal connection between stress, GC activity, and negative impacts on gonad tissue morphology and function.

Given the apparent sensitivity of the bat testes to acute restraint and a concomitant absence of change in steroidogenic enzyme expression, the overall impacts of stress on gonadal cell health are not immediately clear. We detected transcriptional upregulation of two key signaling molecules – Bax and cytochrome *c* – involved in the receptor-mediated apoptotic pathway in the testes of *E. fuscus*. Bax, a member of the bcl-2 protein family, is one of the core apoptotic pathway regulators that interacts with mitochondria and mediates outer membrane permeability (Westphal et al., 2014). An increase in membrane permeability leads to the subsequent release of cytochrome *c* from the mitochondrial matrix, enabling a series of downstream caspase activation steps and cell death (Santucci et al., 2019). While we did detect increases in *Bax* and cytochrome *c* mRNA expression, we did not find significant differences in the percentage of TUNEL-positive cells in the testes of stress-treated bats compared with controls. Because TUNEL is a method that detects DNA fragmentation, a molecular process that would occur only at late stages of cell death, it is possible that we were not able to capture the expected response within the 60 min of our protocol. Interestingly, we found a tendency for control bats to exhibit a greater total percentage of TUNEL-positive cells in their testes. These data – while contradictory to our predictions – may reflect a higher degree of cell turnover, and thereby a higher degree of gonadal activity, within the testes of control bats compared with those that experienced stress given that stressed animals decreased seminiferous tubule diameter and apparent testosterone production. Programmed cell death (i.e. apoptosis) has been shown to be an important component of ‘normal’ spermatogenesis, suggested as one mechanism of maintaining homeostasis and correct gonad function in the testes (Porcelli et al., 2006). Shaha (2007) found that male germ cell death occurs through both death receptor-mediated and mitochondrial pathways of apoptosis and – under healthy conditions – between 50% and 75% of developing germ cells undergo cell death before reaching maturity (Dunkel et al., 1997; Wang et al., 2006). Future studies could couple apoptotic labeling with a marker cell proliferation (such as PCNA), which could help describe the overall cellular dynamics. A combination of testes tissue or isolated cell culture and flow cytometry (Annexin V) could also serve as a valid method for understanding this better but would lack the resolution of seeing which region or cell types within the gonads may be affected.

The upregulation of pro-apoptotic gene expression for Bax and cytochrome *c* was a surprising finding given the short duration (60 min) of our stress manipulation. There is a precedent for pro-apoptotic effects in the gonads following longer periods of stress. For example, female mice restrained for 24 h displayed increased CRH in the ovaries, accompanied by decreased IGF-1 and upregulation of Bax mRNA expression (Liang et al., 2013). Additionally, chronic stress can cause increases in plasma GCs, decreases in testosterone and an increase in the frequency of apoptotic Leydig cells in rat testes (Chen et al., 2012), as supported by the findings of Gao et al. (2003), where *in vivo* Leydig cell treatment with GCs resulted in apoptosis. Additionally, there is a link between intra-testicular RFRP and upregulated gonadal apoptosis. In mice, *in vitro* treatment of testes with RFRP was seen to cause dose-dependent increases in caspase-3 and PARP peptide cleavage indicative of receptor-mediated apoptosis signaling (Anjum et al., 2014). The fact that testes health and function may be suppressed without the direct influence of circulating gonadotropin signaling suggests that GCs are likely acting directly on the gonads to downregulate cellular maintenance and sex steroid synthesis with potentially deleterious impacts on overall tissue health. We know of only one study in rats (Juárez-Rojas et al., 2015) that found a single 15 min cold immersion stress treatment was sufficient to increase the percentage of apoptotic germ cell detection via TUNEL analysis at 1 h after exposure, persisting through to 24 h. The same study also detected significant increases in testes Bax and cleaved caspase-3 and -8 proteins via western blot. We believe this paper supports our finding that acute stress has the potential to rapidly upregulate pro-apoptotic signaling; however, it is important to note that they did not use a restraint stress protocol and did not find a significant decrease in plasma testosterone concentrations in their animals. Thus, while work in other model systems supports the pro-apoptotic effects we observed, the rapidity of the multi-level response we report here – from brain to gonads and circulating hormones – in bats is a novel finding and an exciting avenue for research. Future work should continue to explore gonad function and fertility in female and male bats, identifying details of the underlying cellular biology mechanisms and time frames for recovery before we can make broad claims regarding the sensitivity of bat reproductive physiology and fertility to stressors.

### Conclusions

Stress can lead to the inhibition of reproductive physiology and behavior across a wide array of taxa and it is likely that the specific eco-physiological context a stressor is experienced within distinctly influences the neurobiological and gonadal responses of the organism (Bentley et al., 2017). Collectively, our findings indicate that the brain and the testes in *E. fuscus* respond to acute restraint stress with significant declines in circulating plasma testosterone and potential rapid apoptotic consequences in the gonads coupled with an increase in local RFRP and GR expression. The short time frame of the gonadal response in *E. fuscus* is unprecedented in mammals, suggesting that bats are highly sensitive to acute stressors. As such, our data have implications for conservation management of bat roosts and hibernacula, especially in terms of disturbance. Finally, because stress responses are known to vary temporally, including seasonally, it is important that future studies examine the effects of acute stressors on the HPG axis across the seasons. Temperate, seasonally breeding bats such as *E. fuscus* are a particularly appropriate model system for addressing such questions and the unique asynchrony between male and female reproductive investment presents an opportunity for interesting and novel inquiry surrounding life history, and physiological, trade-offs.
